# Maidstone—Acquittal of the Charge of Murder on the Ground of Insanity

**Published:** 1848-07-01

**Authors:** 


### MAIDSTONE SPRING ASSIZES—March 16.

### CHARGE OF MURDER BY DROWNING.—ACQUITTAL ON THE GROUND OF INSANITY BROUGHT ON BY DESTITUTION AND DISTRESS OF MIND.

*Crown Court.—(Before Mr. Justice Coleridge.)*

Mary Sweetlove, aged 37, married woman, was indicted for the murder of her infant child, by drowning him at Sandwich.

Mr. Rose prosecuted, and Mr. T. Chambers defended the prisoner.

The prisoner was the wife of a baker at Sandwich, and the mother of five children. It would seem that they were in very bad circumstances, and on two or three occasions, the whole family had been admitted to the Union workhouse. In the month of December, last year, it appeared they were reduced to a state of great destitution and misery, the prisoner not having the means of paying for a lodging or providing food for her family; and on the 18th of December she was informed by the landlord that he could not allow her to remain in his house any longer, and that she must provide herself with another place of refuge. Upon this being intimated to the prisoner, it appeared that about the middle of the day she went out, taking with her the youngest child, and in a short time she returned to the public-house, but unaccompanied by the infant. About half-past one o'clock in the afternoon of the same day, a labouring man who was returning to Sandwich, having occasion to pass a small stream, known as the Dyke, saw the body of an infant in the water, and he took it out, and conveyed it to the nearest cottage, whence a medical man was sent for, who used the necessary remedies to restore animation in such cases, but without success. This infant was speedily identified as that of the prisoner, and some persons immediately proceeded to the public house where she was staying, and on her being asked what she had done with her baby, the prisoner replied that she had thrown it into the dyke, and gave as a reason for doing so that the child cried as she was passing the place. The witnesses stated that at this time the prisoner appeared in a wild and excited state, and one of them said that for some time before the melancholy transaction, the prisoner presented the appearance of a lost woman, and she was of opinion that her mind was affected. Mr. Emmerson, the surgeon, who saw the prisoner immediately after the affair, also expressed an opinion that on the day the child was found in the water the prisoner was in such a state of mind as to be unconscious of her actions. It appeared that after the prisoner had been committed to gaol, she sent for Mr. Aris, the governor of the prison, and expressed a wish to make a communication to him; and after he had cautioned her that what she stated would be used as evidence against her, she made a statement, which he reduced to writing, to the effect that on the day in question her landlord had told her that he could not allow her to remain any longer in his house, as she had no money to give him, and that if she did not provide herself with another lodging in two hours, he should turn her and her family into the street. She said, that upon this threat she went out with her child, and having no home, or friends, or any means of support, she was driven almost to desperation, and was not conscious of what she did, and could not tell how the child got into the water—whether she laid it on the bank and it rolled in, or whether she had herself placed it in the water.

Mr. T. Chambers addressed the jury on behalf of the prisoner, commenting in forcible terms upon the destitute and wretched condition to which she was reduced, and urging that she had been driven to despair and madness, and was, in consequence, not in such a state of mind as to render her criminally responsible for the dreadful act she had committed.

Mr. Justice COLERIDGE summed up, and

The jury, after a very short deliberation, returned a verdict of *Not Guilty*, on the ground of insanity.

The prisoner was ordered to be detained during her Majesty's pleasure.

---

We append the following judicious observations, (having reference to the two previously reported cases,) which we extract from the "Medical Gazette," for March 24, 1848:—

"We elsewhere publish the reports of two trials, in which females were charged with the murder of their children. In both instances they were acquitted on the plea of insanity. All who read the details will agree with us that the verdicts were very proper, and that, under the circumstances, it would have been impossible to have returned a verdict of guilty against either party. The two cases, however, are singularly contrasted in the fact, that where the plea was, medically speaking, of a strong and indisputable kind, the judge most unnecessarily, as we think, went out of his way to pass a severe censure on the medical witness, because he stated that, in his opinion, the prisoner was insane; while in the second case, where, under a harsh view of the facts, there might have been room for cavilling at the medical evidence it was admitted without opposition or remark.

"It is on the first of these cases that we propose to offer a few observations. A female was delivered of a child on the 10th of December. About a week afterwards, symptoms of puerperal mania appeared, and Mr. Bell, of Felstead, her medical attendant, ordered that she should be watched, and that she should not be allowed to have the child. On the 23rd December, in the absence of the attendants, she induced her daughter to bring the child to her, and subsequently requested her to give her a razor, with which she almost immediately afterwards cut the throat of her infant. The prisoner appeared quite calm and collected after the occurrence. She admitted that she had destroyed the child, and that it was what she had all along intended to do. The facts deposed to by Mr. Bell and the female attendants of the prisoner, left no doubt that the prisoner was labouring under an attack of puerperal mania at the time the act was perpetrated.

"The medical witness properly admitted, in answer to the judge, that the prisoner might have known that she was going to kill the child, but that she acted under a *sudden* and uncontrollable impulse. Lord Denman objected to the term 'sudden,' because the prisoner had deliberately asked for her child, and had suffered a quarter of an hour to elapse before the razor was asked for, with which she destroyed it. Mr. Bell then stated, as every professional man, acquainted with the subject of puerperal insanity, would have stated—that 'the act was committed under an uncontrollable impulse acting upon a mind previously diseased,'—a conclusion which irresistibly flowed from the premises.

"Lord Denman, in his address to the jury, is reported to have expressed an opinion 'that the judgment of the medical gentleman *had been very rashly formed!* How,' said his Lordship, 'could one person dive into the mind of another, and express an opinion with regard to its being in an unsound state, when there was no evidence of any alteration of conduct, or any circumstances in the case to show alienation of mind?' We do not see how this statement can be reconciled with the evidence of the medical witness, that 'the prisoner's eyes were vacant and wild, and her countenance haggard;' or with that of the women who watched her during her illness, and who deposed 'that she frequently seemed as though her mind was roving, and often said that she knew she was going to die, and, as she was certain she should go to hell, it might be as well first as last; with other incoherent expressions.' The charge of rashness against a highly respectable medical practitioner, in forming his opinion from such facts as these, was surely not warranted under the circumstances. The objection to the use of the word 'sudden' was wholly uncalled for, as the state of puerperal insanity is quite compatible either with a *sudden* or *deliberate* intention to destroy the child. The real point to be considered, however, was not whether the intention to kill had been slowly or deliberately formed, but whether the impulse which led to the act was or was not *controllable.* All practitioners would answer, we believe, with one voice, as Mr. Bell answered on this occasion, that the unfortunate prisoner, even if aware of the nature of the act, took the life of her child under a state of mind in which her will was powerless to control her conduct.

"'How could one person,' asks the learned judge, 'dive into the mind of another?' The obvious answer to this question is, that unless all really insane persons are held responsible for their acts like sane criminals, there is no other way of giving medical evidence respecting their mental condition. If this is to be taken as a sound objection to medical evidence, it appears to us that it would be better to abolish the plea of insanity at once. Because a quarter of an hour had elapsed between the asking for the child and the weapon, his Lordship inferred that the impulse to kill was *not* sudden! He assumed, therefore, that it existed when the prisoner requested to have the child given to her; and this was surely diving into the state of her mind! There might have been some ground for the assumption had a quarter of an hour elapsed between the asking for the *weapon* and the employment of it; but the girl who gave the razor to the prisoner 'had hardly reached the door,' as she states, before the child shrieked. The requesting to have the child was not of itself a proof that the prisoner intended to destroy it. This homicidal feeling may have come on suddenly at a subsequent period, and have been actually excited by the sight of the child.

"The censure of the medical witness for his evidence on this occasion, appears to us to have been quite misplaced. If, as his lordship said, 'there was *no doubt* that the jury would act upon his testimony, then it follows there could be no doubt that his opinion had been *correctly* and not rashly formed. We are no advocates for the getting up of a plea of insanity, in the way in which it is now frequently brought before courts—namely, as a *pis aller* in the defence of criminals when all the other tricks of law fail; but we have seldom met with a case in which the plea was more strongly justified by the facts than in this instance. We can only account for the censure of the learned judge by supposing that it was directed, not so much against the medical evidence in *this* case, as against the capricious introduction of insanity as a defence in other instances in which it should not have been heard of! It is, however, inconsistent with justice that the errors of others should be visited upon one unfortunate witness, who happens, in a case which admitted of no doubt, to give a true and conscientious opinion."

---

